# Predicting high‐performance decathlon career best

**DOI:** 10.1113/EP091921

**Published:** 2025-01-09

**Authors:** Perry Battles, Tyler J. Noble, Robert F. Chapman

**Affiliations:** ^1^ H.H. Morris Human Performance Laboratories Indiana University Bloomington Indiana USA; ^2^ High Performance Department USA Track and Field Indianapolis Indiana USA

**Keywords:** elite athlete, performance modelling, track and field

## Abstract

The decathlon is a 10‐event discipline in the sport of track and field, typically offered only for men at the elite level of competition (heptathlon is the complementary event for women). It is composed of 10 distinct events contested over 2 days. Using event‐specific coefficients, marks are converted to scores, which sum to produce an overall score. Ten events that share some underlying features, but also some disparate features, create a challenge for coaches and sports administrators in identifying potential elite talent and developing it optimally across the career. In this investigation, decathletes were profiled on the basis of keeping performances ≥6400 points and including only those participants who competed in ≥4 years (n=642). Using gamma generalized linear regression models, the first‐season best mark and improvement in each event were related to all‐time career best. Coefficients were compared to infer priorities for elite decathlete selection (using first‐season best marks in each event) and development (using the delta or improvement in each event). These data show that the most crucial event for identifying an athlete with a future potential elite all‐time career best is the pole vault, followed by the javelin throw, long jump and shot put. For optimal career development, the data suggest athletes obtain the most return by focusing on improvement in the pole vault and long jump.

## Abstract (highlights)

- **What is the central question of this study?**
  What are the most important event strengths to look for in selecting and developing an elite track and field decathlete?
- **What is the main finding and its importance?**
  Using a gamma generalized linear model, this investigation determines that first‐season performances in the pole vault, javelin throw, long jump and shot put are crucial for identifying athletes with elite potential. To develop a decathlete with elite potential over a career, an approach that emphasizes improvement in pole vault and long jump is indicated to facilitate optimal career performance.

## 1 INTRODUCTION

The decathlon is a 10‐event discipline in the sport of track and field contested over 2 days of competition. The events consist of (in order) the 100 m, long jump (LJ), shot put (SP), high jump (HJ) and 400 m (all on day 1), and the 110 m hurdles, discus throw (DT), pole vault (PV), javelin throw (JT) and 1500 m (day 2). The athlete's performance mark for each individual event earns points based on a scoring table [International Association of Athletics Federations 2001 (Reprinted June 2016)], and point contributions from all 10 events are added for an overall score.

Athletes do not normally become decathletes as their first‐choice event in the sport of track and field. Commonly, US collegiate or international club coaches will encourage athletes with moderate (but often not elite) ability across a wide range of events to try the decathlon, because they probably could not achieve competitive national or international excellence in any one event. Conversely, there are examples of multisport track and field athletes, including decathletes, who are successful at the highest level of competition in individual events, implying that outsized performance in specific disciplines and success in the multi‐events might not be mutually exclusive. The challenge for coaches in identifying and training decathletes for national‐ or international‐level success is twofold: (1) to determine which individual events and what baseline level of ability in those events are predictive of potential future success in the decathlon; and (2) from a physiological and skill development perspective, once a potential first‐year decathlete is identified, which events are the most crucial to improve in to progress to an elite level in the decathlon. This latter point is important for the coach, because the physiological and technical components of the different events are not always complementary, and determining how to plan logistically for and allocate training time for 10 events can be a challenge.

The presence of multiple events with some level of shared underlying athletic abilities has focused much of the inquiry to date concerning the decathlon, with a goal of resolving these events into relevant groups and determining which of them matter the most for overall performance (Dziadek et al., 2022; Park & Zatsiorsky, 2011). Intuitively, events are grouped by technique similarity and physiological demand: the sprints (100 m, 110 m hurdles and 400 m), the jumps (LJ, HJ and PV) and the throws (SP, DT and JT), with the 1500 m occupying its own category as a short endurance event. However, analysis of marks in the 10 events has determined that the boundaries between them are not so clear cut. Walker and Caddigan (2015) observed that, although correlations between events were generally higher within functional groupings (sprints, jumps and throws) than without, the PV did not appear to be very associated with the other jumps, and the 1500 m, if grouped with the other running movements, was not correlated with the other members of its functional group. Additionally, the groupings might not be consistent across the entire career. Dziadek et al. (2022) found that, in early‐stage decathlon, the first principal component factor loadings indicated groupings such as speed/strength (400 m, 110 m hurdles, LJ, SP and DT), mixed sprint/jump/throw/endurance (JT, 1500 m, 100 and HJ) and another mixed jump/throw group (PV, HJ and SP). This contrasts with loadings during later‐stage decathlon, which revealed the groupings of sprints (100 m, 400 m, 110 m hurdles and LJ), strength/endurance (DT, SP and 1500 m), jumps/throws (HJ, JT and LJ) and sprint/jump/throw/endurance (PV, 1500 m, 110 m hurdles and JT) (Dziadek et al., 2022). These groupings not only disagree with one another but also with the expected associations that follow from the physiological demands of the disciplines (e.g., the hurdles finding themselves in the same group as the 1500 m in late‐stage decathlon). Although there are expected inconsistencies in such groupings owing to technical factors and even training logistics, these data beg the question of whether there are alternative ways to analyse the contribution of the events to the overall score beyond correlation or an analysis of the transfer of training via grouping.

Although determining relevant event groupings is valuable for understanding transfer of training between disciplines, it is not obvious how it might help to make predictions concerning whether an athlete can or will be successful over the span of their career. It is generally agreed that success comes down to a combination of initial talent and making outsized improvements in particular events, but evidence is conflicting concerning what those events are. Additionally, the unique scoring equations for each discipline might bias evaluation of emerging athlete potential based on event area strengths (e.g., the jumps, throws, sprints and the 1500 m run). These equations attempt to create common ground between the events for scoring purposes, because an improvement of 1 m in the JT is not nearly as meaningful a performance increment as an improvement of 1 m in the SP, for example. However, even with the help of the scoring equations, it is not obvious that a 50‐point improvement in one event is achievable with the same investment of time and effort as an equivalent improvement in another event. The relationship between the score achieved in an event and the investment required to achieve it is made even more opaque by the presence of shared underlying features among the events, the strengths and weaknesses of individual athletes, and the balance of technical and physiological elements for each event.

The aim of this study was to use existing data on elite decathletes to quantify the relationship between an athlete's career overall personal best (PB) and their first‐season best marks (FSB) and improvement in each event (delta), because this can help to provide objective event‐specific measures for predicting emerging athlete potential.

## 2 MATERIALS AND METHODS

### 2.1 Ethics approval

This study used pre‐existing, publicly available data, did not make use of direct measures on human or animal subjects, and therefore did not require ethics approval. This study complied with the *Declaration of Helsinki*.

### 2.2 Data deposition

The raw data and processing pipeline for this article are available at the following address: https://github.com/Battles186/DecathlonCareerBest.git.

### 2.3 Terminology

Event = one of the 10 individual events that make up the decathlon.
Mark = the performance (in time or distance) achieved by the athlete in one of the events within the decathlon.
Score = each individual event mark from the decathlon competition is converted to a score, and each of the 10 scores is added to determine an overall score for the decathlon, which determines the placings within a competition.

### 2.4 Inclusion criteria

Competitive performances for high‐level men's decathlon from the years 2001–2022 were obtained from a publicly available database (World Athletics). Only overall scores of >6400 points were kept, because this denoted a subjectively determined minimum value that would yield a realistic chance of becoming elite (Supplementary Material 1). Within this dataset, the inclusion criterion was that athletes competed on record for at least four seasons. This narrowed the sample to only those athletes who were chronically relevant from a competition standpoint and left an overall athlete count of 642.

### 2.5 Data extraction

Prior to extraction, the data were preprocessed by converting marks in the 100 m, 110 m hurdles, 400 m and 1500 m from times to speeds, such that a larger point reward in any event always corresponded to a larger mark and an improvement in any event would always correspond to a positive value. Data extraction consisted of identifying the FSBs and all‐time event best marks from the raw data, such that each observation in the resulting dataset consisted of an athlete profile of FSBs and all‐time best performances in each event. Performance deltas were then computed by subtracting the FSB mark from the all‐time best mark for each event. The highest overall meet score for the decathlete (PB) was also extracted.

### 2.6 Statistics

Before model fitting, the performance delta variables were transformed using Box–Cox scaling (Box & Cox, 1964). Values of λ from −20 to 20 were explored in increments of 0.1, and the λ among those corresponding to the highest calculated log‐likelihood was selected for use in the transformation of each variable. This λ value was also rounded to the second decimal place. This resulted in λ values between 0.3 and 0.6 for each feature.

Given that Box–Cox scaling does not handle negative values by default, a correction was used for the delta features to ensure there would not be negative values provided as input to the scaler. This correction factor was equal to zero if all the values in the feature were above zero, or equal to the absolute value of the smallest element in the feature plus 0.01 if there were elements at or below zero. This resulted in a correction of 0.01 for each delta feature.

Following this Box–Cox transformation, all predictors were subjected to robust scaling. This additional level of scaling was selected for two reasons: (1) it has minimal assumptions in comparison to other modes of scaling; and (2) it is more robust to outliers compared with other common forms of scaling. Owing to the nature of studying elite sports, outliers are often the most important observations in any dataset. As such, care should be taken not to distort the data or needlessly eliminate extreme observations while still allowing statistical models to provide grounds for inferential claims.

Before model fitting, tests for collinearity were performed on the transformed data. It was found that only two features were affected (r≥0.7): $\mathrm{FS}B_{\mathrm{DT}}$ and $\mathrm{FS}B_{\mathrm{SP}}$.

Three generalized linear models were fitted to the data using FSB and delta marks as covariates and PB as the outcome variable. The first model was fitted with a log link, the second with the inverse link [the canonical linking function for gamma generalized linear models (Dunn et al., 2018: table 6.4)], and the third model was fitted using the identity link function. Visual inspection of residuals was used to assess model appropriateness and to verify model assumptions.

Subsequent to model fitting, influential outliers were identified using a panel of tests. These included the DFBETAS and DFFITS for each model variable, the covariance ratio, and Cook's distance, as described by Belsley et al. (1980) and Cook and Weisberg (1982).

Model coefficients for FSB and delta values were rank ordered by magnitude to compare and contrast their effects on PB. Subsequent to fitting, Spearman's ρ was used to compare the ordering of event coefficients to assess agreement between the models. For all statistical tests conducted in this investigation, α was set to 0.05.

Importantly, this is an exploratory investigation by nature. Although the comparison of many coefficients across three separate models will certainly inflate familywise error wildly, it is in the nature of exploratory investigation often to forego a hypothesis and compare many factors that might influence the outcome of interest (Sullivan & Feinn, 2021). This is especially true in light of the fact that investigations into the decathlon have found either inconsistent or conflicting results within themselves (Dziadek et al., 2022) or between one another (Van Damme et al., 2002; e.g., Kenny et al., 2005). As such, we performed pairwise comparisons to explore the relationships between model predictors more comprehensively.

To compare model coefficients, a bootstrapping technique was applied to the difference in estimates of two coefficients. For each of 1000 bootstrap replicates of the data (sans outliers), a model with the appropriate link function was fitted to the bootstrapped sample, relating all predictors to the outcome. The difference for the pair of coefficient estimates in question was extracted and used in the construction of a 95% confidence interval. If the confidence interval of the difference between the two coefficient estimates crossed zero, there was no significant difference at α = 0.05. If the lower bound of the confidence interval was above zero, this constituted a significant difference, in which the first coefficient in the pair was greater than the second. If the upper bound of the confidence interval was below zero, this indicated that the first coefficient was significantly less than the second. This process was repeated with 1000 boostrap replicates for each coefficient comparison. Bias‐corrected accelerated confidence intervals were used for the comparison of model coefficients.

Bootstrapping confidence intervals for the model coefficients were used in order to permit direct, testable comparisons between model coefficients in terms of their effect on estimates of the outcome variable. This approach removed the constraints or limitations of other methods; linear hypothesis testing, for example, permits direct coefficient comparisons but introduces the constraint of testing for equality rather than directional inequality. Given that one of the aims of this investigation was to establish ranked priorities for decathlete selection and development, a non‐directional hypothesis test was not well suited to this purpose. Additionally, because the generalized linear model was used, there were few or no established conventions surrounding hypothesis tests comparing two coefficients within a generalized linear model. For this reason, more standard methods, such as Welch's *t*‐test, were not used. Finally, with 1000 replicates per comparison, it seems that spurious results would be unlikely.

One important consequence of this bootstrapping approach to coefficient comparison is that a row‐versus‐column comparison can have a different outcome from a complementary column‐versus‐row comparison of coefficients, especially when the confidence interval for the comparison in question is very close to zero (e.g., in the inverse link model delta coefficients, the PV was significantly more influential than the 110 m hurdles on one axis but not on the other). This affected the interpretation of differences between the HJ and 400 m, the HJ and JT and the 1500 m and 400 m among the FSB coefficients, in addition to comparisons of the PV and 110 m hurdles and the 1500 m and JT among the delta coefficients.

Importantly, although it is true that a 100×α‐level confidence interval that does not contain zero corresponds to a significant difference at P≤α and has an accompanying P‐value, the volume of comparisons performed in the present study means that there would be 270 such P‐values reported. As such, these have not been presented in the text, but are indicated in figures using colour‐ and hatch‐coding to signify significant differences.

## 3 RESULTS

### 3.1 Model appropriateness

#### 3.1.1 Standardized deviance residuals

The plot of the standardized deviance residuals against the log of the fitted values did not appear to display any strong discernable trends for either the log link or identity link models. The plot for the inverse link model, however, appeared to display a trend.

#### 3.1.2 Linear predictor versus working residuals

A plot of the linear predictor against the working residuals demonstrated high model appropriateness with respect to the data for all three models.

#### 3.1.3 Partial residual plots

Partial residual plots further confirmed model appropriateness, with errors for all features following the ideal relationship. Those that deviated the most from this relationship were the delta values for the 110 m hurdles, 400 m, SP and 1500 m.

### 3.2 Model coefficients

Each of the three distinct models was refitted following outlier detection and removal. Once this process was complete, coefficients for all first‐year event marks and career deltas were significant at *P* < 0.0001 in all three models, with the exception of delta_100_, which was significantly related to the outcome at *P* < 0.001 in the log link model, *P* = 0.001 in the inverse link model, and *P* < 0.001 in the identity link model.

### 3.3 First‐season bests

**FIGURE 1:**
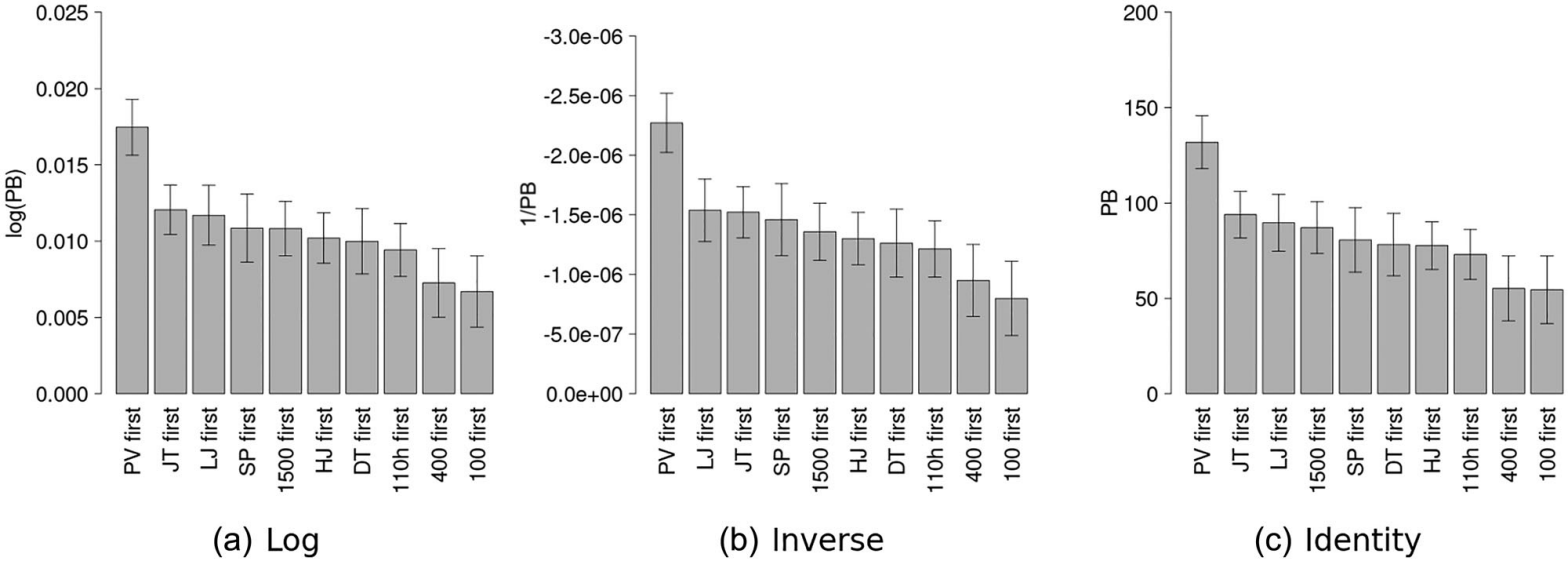
Absolute magnitudes of FSB coefficients, sorted from highest to lowest. Error bars indicate a 95% confidence interval. Abbreviations: DT, discus throw; FSB, first‐season best marks; HJ, high jump; JV, javelin throw; LJ, long jump; PV, pole vault; SP, shot put; 100, 100 m; 110h, 110 m hurdles; 1500, 1500 m; 400, 400 m.

Figure 1 shows the FSB coefficients, sorted by absolute magnitude, for each model. The models disagree slightly about the positions of the HJ, SP, 400 m and 100 m. The PV is ranked highest across all three models, followed by either the LJ or the JT depending on the model. There was a high degree of correspondence between the log link model and the inverse link model (Spearman's ρ = −0.995, *P* ≤ 0.05) and between the identity link model and the inverse link model (Spearman's ρ = −0.982, *P* ≤ 0.05). The same was true of the log and identity link models (Spearman's ρ = −0.989, *P* ≤ 0.05).

**FIGURE 2:**
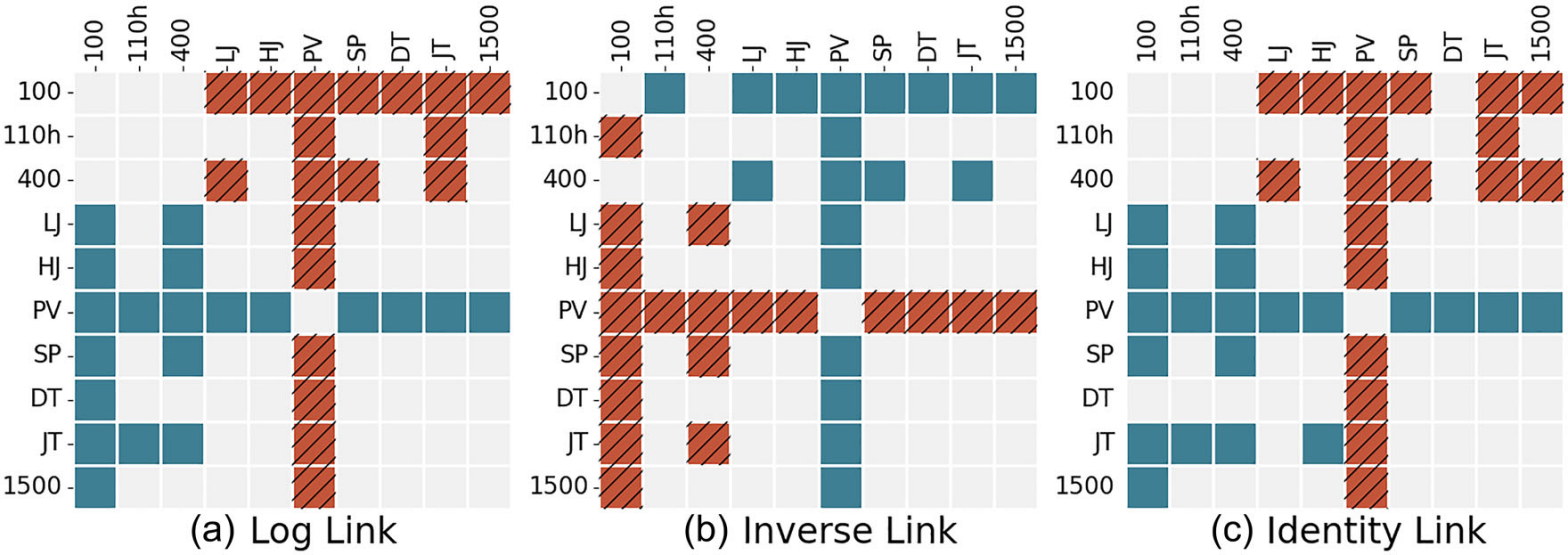
Matrix diagram showing significant differences between FSB coefficients. A hatched orange square indicates that the entirety of the bootstrapped confidence interval of the difference between the row coefficient and column coefficient is below zero, indicating the column coefficient is greater. A teal square indicates that the entire confidence interval is above zero, meaning that the row coefficient is greater. A grey square indicates that this interval contains zero, which means that neither one is significantly greater than the other. Abbreviations: DT, discus throw; FSB, first‐season best marks; HJ, high jump; JV, javelin throw; LJ, long jump; PV, pole vault; SP, shot put; 100, 100 m; 110h, 110 m hurdles; 1500, 1500 m; 400, 400 m.

Figure 2 shows which coefficients are significantly different from the others at P≤0.05 according to the bootstrapped coefficient comparison method. For example, the coefficient for the PV was significantly greater (P≤0.05) (or smaller in the inverse model, given the nature of the linking function; subsequent indication of this difference will henceforth be omitted) than all others across the three models (P≤0.05). Additionally, the coefficients for the LJ, PV, SP and JT were all significantly greater (P≤0.05) than the coefficients for the 100 m sprint and 400 m sprint in all three models. The coefficient of the JT was significantly greater (P≤0.05) than that of the 110 m hurdles in the log and identity link models. Additionally, considering the 100 m sprint alone, the LJ, HJ, PV, SP, JT and 1500 m all had coefficients that were significantly greater than that of the 100 m (P≤0.05) across all three models. The DT is also included in this group if only the log and inverse link models are considered.

Several events were not significantly different from one another; these consist chiefly of events that share the same group (e.g., 100 m and 400 m or DT and JT). For further examples, see Figure 2.

### 3.4 Deltas

**FIGURE 3:**
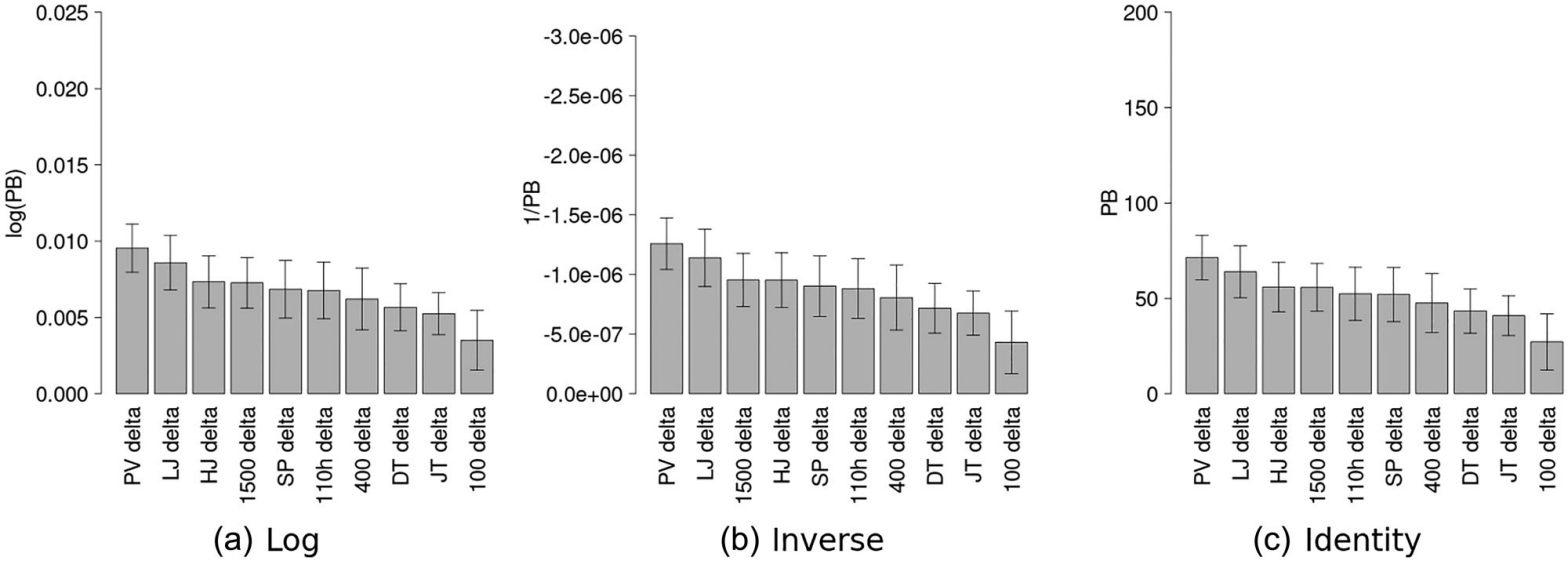
Absolute magnitudes of delta coefficients, sorted from highest to lowest. Error bars indicate a 95% confidence interval. Abbreviations: DT, discus throw; HJ, high jump; JV, javelin throw; LJ, long jump; PV, pole vault; SP, shot put; 100, 100 m; 110h, 110 m hurdles; 1500, 1500 m; 400, 400 m.

Figure 3 shows the delta coefficients, sorted by absolute magnitude, for each of the models.

**FIGURE 4:**
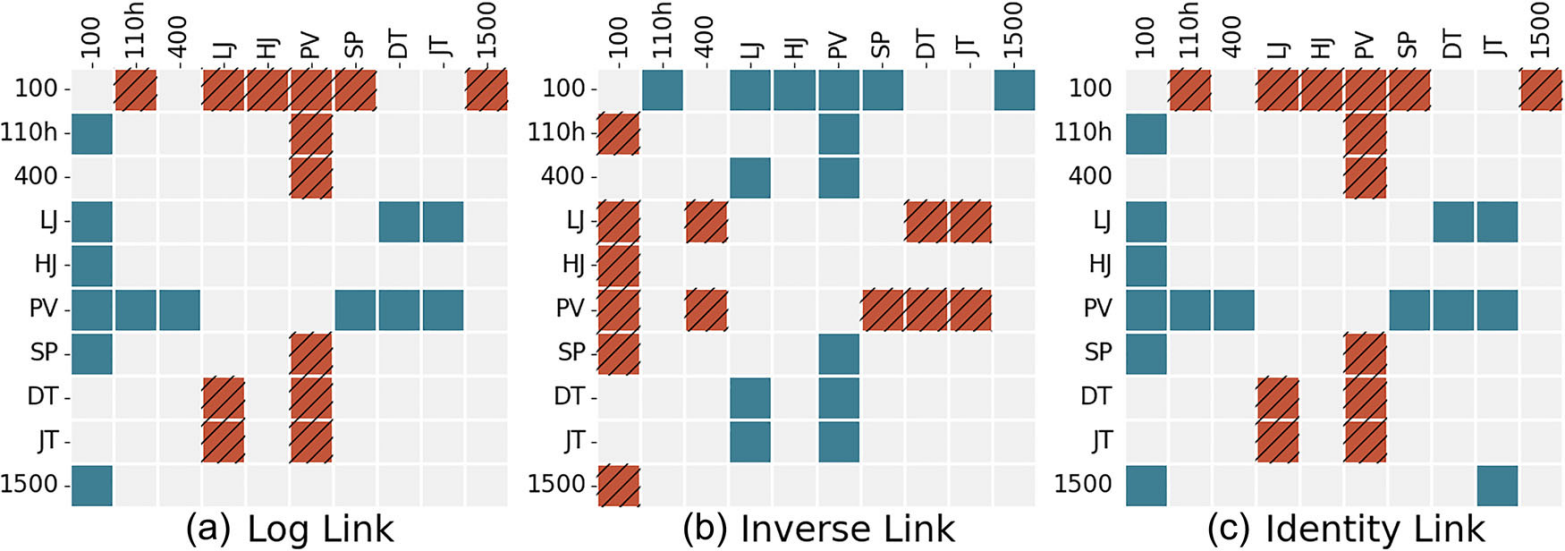
Matrix diagram showing significant differences between delta coefficients. A hatched orange square indicates that the entirety of the bootstrapped confidence interval of the difference between the row coefficient and column coefficient is below zero, indicating the column coefficient is greater. A teal square indicates that the entire confidence interval is above zero, meaning that the row coefficient is greater. A grey square indicates that this interval contains zero, which means that neither one is significantly greater than the other. Abbreviations: DT, discus throw; HJ, high jump; JV, javelin throw; LJ, long jump; PV, pole vault; SP, shot put; 100, 100 m; 110h, 110 m hurdles; 1500, 1500 m; 400, 400 m.

Figure 4 shows the results of the bootstrapped coefficient comparisons. There were minimal differences between the three models. The only difference between the results for the log link and identity link models was in a comparison between the 1500 m and the JT, which indicated that the JT was significantly less influential than the 1500 m in the identity link model (Figure 4c). This difference also held between the inverse link model and the other two models, in addition to the fact that the LJ was significantly more influential than the 400 m in the inverse link model (Figure 4b). There was also a mismatched comparison in the inverse link model, in which the PV was significantly more influential than the 110 m hurdles along one axis but not the other (Figure 4b).

Apart from these differences, the patterns found in the delta coefficients are generally similar to those of the FSB coefficients. The PV had a significantly greater coefficient than all of the sprints (with the exception of the 110 m hurdles in the inverse link model on one axis of comparison) and all of the throws (P≤0.05), but not the other jumps or the 1500 m (Figure 4). The LJ had a significantly greater coefficient than the 100 m, DT and JT at P≤0.05 across all three models. Finally, the 100 m had a significantly smaller (P≤0.05) coefficient than the 110 m hurdles, LJ, HJ, PV, SP and 1500 m across all three models (Figure 4).

## 4 DISCUSSION

The decathlon coach Zdenek Váña concluded his 2003 presentation on the success of his team, which included some of the most elite decathletes of all time, as follows:

I am just an ordinary coach, I started coaching youths and then the sprints and hurdles. I was very lucky and fortunate with the choice of athletes, who wanted to work hard so we made it to world‐class performances and breaking the mythical 9000 point barrier in the decathlon, the royal event of track and field.

(Vana, 2003: p.30)

Váña emphasizes his good fortune in selecting excellent athletes early on and attributes their subsequent success to this. Although there is certainly more to the achievements of his cohort than simply choosing the best athletes early on, there is no doubt that identifying excellent talent played a substantial role in their achievement. Váña credits this to his good fortune; however, for the coach and sports administrator looking to identify and develop elite talent in this complex discipline, chance cannot be relied upon to facilitate success. Instead, identifying the factors that predict long‐term career success and quantifying them can help to optimize talent identification and development.

In the 10‐part track and field decathlon, the presence of multiple events with shared underlying characteristics makes the prediction of future performance a multidimensional problem. Additionally, given that the decathlon is an event that draws talent from many other disciplines, it is precisely the ability to identify outlier potential based on individual event performances that is crucial. Once talent has been selected, the next question that follows is how to invest time and effort to maximize the odds of producing a champion. Give that these questions have not yet been addressed thoroughly with predictive mathematical modelling, we used a tool that is novel in this research niche to predict long‐term career achievement in the track and field decathlon. This investigation builds upon past research, which has established associations among the events, attempted to identify trade‐offs among them, and profiled elite performers. The data presented here have determined that the PV and, to a much lesser extent, the JT are the two factors that distinguish decathletes with elite career potential from their peers, with the LJ and SP following as secondary priorities. These data also indicate that improvement in the PV and the LJ set elite decathletes apart from their peers.

### Previous research in the decathlon

Cox and Dunn (2002) performed a multifaceted analysis of the decathlon events, with the goal of identifying groupings among them and determining whether the decathlon was truly fair (in the sense that it rewarded excellence in each of the events equally). Using data from 1991, 1993, 1995, 1997 and 1999, they initially performed cluster analysis, obtaining the following event groupings for the combined data across all years: (1) 100, 400 m, LJ and 110 m hurdles; (2) SP, DT, JT and PV; and (3) HJ and 1500 m. The groupings they found point to associations between the events that group the sprints together (especially when considering the LJ as a sprint) and do the same for the throwing events. In addition to their cluster analysis, Cox and Dunn investigated potential advantages for particular events owing to higher variance among their scores, finding that the three events with the lowest variance were track events (400 m, 100 m and 110 m hurdles), whereas the field events, particularly the PV, had higher variances.

Finally, Cox and Dunn (2002) used a cumulative sum to assess event fairness. By computing the proportion of overall score attributable to each event and subtracting the mean, profiles for each event can be established showing the pattern from the highest‐ranked decathletes to the lowest. On the basis of this analysis, Cox and Dunn (2002) found that, although there are no individual events that consistently demonstrate the favourable ‘mountain’ profile or the unfavourable ‘valley’ profile consistently year to year, the favourable shapes all occur among the field events, whereas the unfavourable shapes all occur among the track events.

Overall, the results of Cox and Dunn (2002) indicate that the events cluster on the basis of their technical and physiological features and that the field events are favoured over the track events. This agrees with the data presented here, which point to the prevalence of PV, JT, LJ and SP in long‐term decathlon achievement. It is possible that the underlying similarities mentioned above give rise to the prominence of these events via the transfer of training. If improving in one of these disciplines increases performance in another in some meaningful way, it seems logical that these disciplines would become the most common areas of emphasis for successful athletes. Although it is not truly possible to infer on the basis of the present data what these underlying features are, there is evidence that they exist (Cox & Dunn, 2002; Park & Zatsiorsky, 2011).

Park and Zatsiorsky (2011) also used principal component analysis, revisiting the 1962 analysis by Zatsiorsky and Godik (1962). They concluded, on the basis of their analysis, that ∼70% of decathlon performance can be attributed to three latent factors: sprinting performance (100 m, 400 m, 110 m hurdles and LJ), throwing/jumping ability (JT, SP, DT, HJ and PV) and endurance (1500 m). These results agree, to some extent, with the data presented here, given that the first factor contains the LJ, which was an important event among both the FSB and delta features. However, the PV, which was by far the most valuable event in the present analysis, occurred in only the second principal component. This component, however, did also contain the JT, which would be considered a training priority in the present analysis. Hence, there is moderate agreement between the results of Park and Zatsiorsky (2011) and the present investigation if one considers the amount of total variance that an event contributes to be indicative of its importance.

Pavlović and Idrizović (2017) used factor analysis to look at associations between the event performances of 10 world record holders and inferred from their results that there were three essential elite decathlete archetypes: the ‘jumper‐thrower‐runner’ (high factor loadings of PV, HJ, DT, 400 m and SP), ‘runner‐sprinter’ (100 m and 110 m hurdles) and ‘jumper‐thrower’ (LJ and 1500 m). Pavlović and Idrizović (2017) conclude that the areas of greatest strength for elite decathletes are the LJ, 110 m hurdles, 100 m and PV, with the weaker areas being the HJ, JT, SP, DT and 1500 m.

Pavlović et al. (2020) then addressed an adjacent topic by comparing the best decathlon results of several elite athletes with their best results in each individual event. They concluded that decathletes tended to outperform their decathlon bests significantly with their individual bests in each event, particularly in the HJ, 110 m hurdles, DT, PV and 1500 m. The data of Pavlović et al. (2020) suggest the possibility that elite decathletes tend to perform well in the events that will be affected by the fatigue from the first day, as indicated by the high discrepancy in performance or ‘performance reserve’ that they exhibit.

Finally, Dziadek et al. (2022) expanded upon these analyses by introducing the career stage. Arguably, their analysis was influenced by differing sample sizes between stages but revealed the potential for varying associations between events at different levels of decathlete advancement. Dziadek et al. (2022) conclude that speed and strength are the two biggest pillars of decathlon performance, a notion that is supported by the genetic data (Ben‐Zaken et al., 2022; Remmel et al., 2023) and by the opinion of at least one elite decathlon coach (Vana, 2003). Our results agree, in part, with this notion, considering that the LJ and JT were among the events whose coefficients were significantly greater (P≤0.05) than those of some others. Our data also strongly point to the prominence of the PV, which contradicts the findings of Dziadek et al. (2022) insofar as the PV was typically part of later principal components that explained only a relatively small proportion of the total variance in their dataset. The same can also be said to some extent of the JT.

### 4.1 First‐season bests

The results of the present study indicate that the PV is by far the best indicator of long‐term achievement in the decathlon. The FSB values that are decidedly less important than the PV but still valuable are the JT, LJ and SP. There is also evidence that the 100 and 400 m sprints are the least impactful indicators of career achievement compared with the other events (Figure 2).

Superficially, this contradicts certain established track and field training principles, which hold that speed is the foundation of performance in the decathlon (Vana, 2003). However, it is possible that elite and non‐elite professional decathletes are similarly advanced in the sprints and that it is the mastery of the other elements that Váña mentions (strength, followed by technique) that sets champions apart. Mastery of the PV could be an indication that the decathlete has already become fast and strong and is now focusing on refining technique, particularly in the most technically challenging events. In this model, such disciplines (e.g., PV) are a proxy for overall decathlon advancement. According to the interpretation above, this could indicate that high performances in the PV are most predictive of high long‐term career achievement because they indicate an emphasis on technical practice that can occur only once strength and speed have been built and technique can solidify. The throws would be a natural second priority here, because high performances in them would indicate that speed has already been established, permitting emphasis on throwing. Conversely, poor performances in the throws might indicate that speed (the earliest stage of development) is still in need of a relatively high training focus, placing the decathlete behind relative to the competition. This would explain why the PV (and, secondarily, the throws) are more strongly influential on predictions of career PB than the sprints. A simpler explanation might be that higher first‐season performance in the PV is simply an indication of prior experience in the event and that this is advantageous in its own right, because it is, anecdotally, one of the most difficult to master.

Finally, it bears mentioning that there were several events that were not significantly different from one another (e.g., DT and HJ, 400 and 100 m in Figure 2). Hence, although there are certain outstanding priorities (such as the PV) and non‐priorities (such as the 100 m), several event pairings exist for which definitive comparisons cannot be made on the basis of these data. This lends support to the notion that the decathlon is an event that effectively rewards performance in each of the 10 disciplines relatively equally (somewhat in contrast to the assertions of Westera, 2006). Hence, although these data indicate that high first‐season marks in the PV, JT, LJ and SP are important for greater long‐term career achievement, they also point to the notion that several of the events are likely to be of similar importance.

### 4.2 Improvement across the career

The priorities for the development of elite talent indicated by our data are somewhat narrower than those for early career selection. The PV and LJ are still prominent, but the SP is significantly more influential only than the 100 m; furthermore, the JT does not have a significantly more influential coefficient than any other event in any model (Figure 4). In agreement with the FSB results, the $\mathrm{delt}a_{100\;m}$ is significantly less influential than the 110 m hurdles, LJ, HJ, PV, SP and 1500 m (Figure 4).

Additionally, much like the FSB coefficients, several of the delta coefficients are not significantly different from one another (e.g., DT and JT, 100 and 400 m, as shown in Figure 4). Hence, there are no clear‐cut statistical grounds to prioritize one over the other; this decision must be made based on the individual strengths, weaknesses and training context of each athlete.

### 4.3 Application of these findings

These results can be interpreted as a function of the career stage of the athletes in question. As noted by decathlon coach Zdenek Váña, there is a sequence of priorities for developing decathletes that begins with speed, followed by strength and ending with technique (Vana, 2003). Given that early‐stage professional decathletes often come directly from the university athletic setting and might not have competed in the decathlon prior to university, it is possible that they are at a level of advancement in which the best decathletes will be more experienced or advancing more quickly, hence already focusing on technique, whereas the less experienced or more slowly advancing decathletes will still be focusing more on speed and strength. This would explain why the PV, a highly technical event, is most important both for first‐season prediction and for development of a high career PB. The secondary prominence of the SP and JT confirms this, because strength is developed before technique in this model. Hence, performance in highly technical events would be indicative of an advanced or well‐developed athlete, followed by performance in the throws. Conversely, athletes who are still concentrating primarily on the sprints might be ‘behind’ for the post‐university career stage from this standpoint.

The data of Cox and Dunn (2002) also support this notion. Three events whose scores have the lowest standard deviations (400 m, 100 m and 110 m hurdles) are in the top four for largest median scores. High variance can be taken as an indication that an event is rewarded unfairly for outsized performances because of a favourable scoring equation but could also indicate that these are events in which the abilities of athletes vary more widely than in others. This suggests that some athletes are much more proficient than others and points to potential asymmetries in the selection and development of the decathlete. It is noteworthy that Zdenk Váña started off coaching youths in sprinting events (Vana, 2003), that he suggests a sequential model of decathlete development that begins with speed as the foundation, and that decathletes are, anecdotall,y often recruited from the ranks of sprinters (Wang, 2017). If these assumptions are made, low variance and high median scores in the sprinting events are a logical consequence, because most decathletes are proficient in them; they would logically derive a higher score from those events than they do from the throwing and jumping events, because they are trained in and recruited from the sprinting events. Furthermore, this foundation in the sprints would mean that there would be few decathletes who lack such proficiency. The other events, however, from which decathletes are not recruited and which are further along in the sequential athlete development model, would exhibit higher variance, because some decathletes would be much better than those who are less advanced and because they might not be as disposed towards high performances in those events owing to their selection from a pool of sprinters. The data presented here also support this, because coefficients for FSB performances in the LJ, PV, SP and JT are significantly (P≤0.05) greater than those for the 100 m and the 400 m, for example.

Alternatively, this could simply be an indication that the field events are favoured, as determined by Cox and Dunn (2002). All the events given priority by the present analysis (PV, LJ, JT and SP) are field events. Contrastingly, the track events appear not to be favoured. This could be attributable to the nature of the attempt structure of the field events, which allows for the best mark among several to be chosen in place of a single performance. Alternatively, although somewhat less likely, there could be mathematical grounds for this apparent imbalance (Westera, 2006). The data presented here provide some statistical indication that improving in one subset of the events is likely to be much better than improving in another and that certain initial event strengths will lead to higher long‐term achievement compared with others. This could be interpreted as support for the argument by Westera (2006) that the decathlon might be mathematically unfair.

There is also a possibility that athletes who tend to excel in certain events are selected preferentially for participation because they are judged subjectively to have greater potential. Contrasting the more mathematically deterministic notions of decathlon achievement of Westera (2006), this view would suggest that judgments about an athlete's potential made on the basis of their performance in certain events might play a prominent role in determining which athletes continue to compete in the decathlon and achieve high career PBs. This could be attributable to increased encouragement and social or financial support or to another socially mediated factor. Although speculative, it is important to retain the notion that long‐term achievement in the decathlon might not be merely a function of skewed scoring coefficients, as suggested by Westera (2006), but could also be a function of other influences that do not depend strongly upon the mechanics of scoring. If this is the case, the models presented here would reflect not only the decathlon scoring coefficients, but also the judgments of coaches, peers and sports administrators.

There is also the confounding factor of the existing training priorities in the decathlon. If coaches, particularly the coaches of elite athletes, have chosen to prioritize certain events, this would suggest that these events would naturally become associated with elite decathlete development. In other words, it might be that elite coaches consistently choose similar training priorities for their athletes and that this shapes the profile of the elite decathlete (as treated by Pavlović & Idrizović, 2017) artificially.

### 4.4 Directions for future research

Importantly, there is a major unanswered question concerning a key component in the strategy of elite decathlete development; namely, the difficulty of improving in each event. For example, it might be that there is tremendous point yield to be obtained from improving only a very small amount in a given event, but that even such a minute improvement is a nearly insurmountable task for the typical high‐level decathlete. In this situation, it would be beneficial to weigh the yield of an improvement in a mark by the feasibility of that improvement. The limitations of the data currently available for the decathlon, which consists only of marks and scores from the competition, do not tell much of the story of the training plan or the allocation of time and effort made in training each discipline. Consequently, this question is one that we have not attempted to answer. However, knowledge concerning the feasibility of a given point score improvement in each of the events would be highly beneficial in developing future elite decathletes.

Another question left open by the present research is why the most prominent events have risen to the top. It could be, as suggested by the data of Cox and Dunn (2002), that there is a larger variation in the scores of those events, which would indicate a greater difference between a high and low performer. It could also be the case, as contended by Westera (2006), that these differences are driven by the scoring equations rather than by physiological or technical features. On the contrary, it could be the case that transfer of training is responsible. Answering this question would permit more informed decisions in training the elite decathlete.

Finally, it is not currently known how much of the associations between high marks in certain events and all‐time career PB are attributable to choices in training rather than physiological, technical or biomechanical factors. Answering this question would help to frame the importance of more innate biological features compared with training choices.

## 5 CONCLUSION

Taken together, these data outline key priorities and non‐priorities for success in the professional decathlon. When selecting decathletes, the PV is likely to be the single most important indicator for choosing future champions, followed somewhat distantly by the JT. Additionally, the LJ and SP are generally more important than 100 and 400 m and can be considered secondary priorities. In fact, the 100 m, for example, is less important than the 110 m hurdles (in one model), LJ, HJ, PV, SP, DT (in two of three models), JT and 1500 m in terms of elite decathlete selection. Once an athlete has been selected, the pathway for development should emphasize the PV and LJ. Overall, the PV and LJ are indicated as the highest priorities for decathlon performance.

## AUTHOR CONTRIBUTIONS

Conceptualization: Robert F. Chapman, Tyler J. Noble and Perry S. Battles. Methodology: Robert F. Chapman and Perry S. Battles. Project administration: Robert F. Chapman. Writing (review and editing): Robert F. Chapman. Data curation: Tyler J. Noble. Formal analysis: Perry S. Battles. Software verification: Perry S. Battles. Visualization: Perry S. Battles. Writing (original draft): Perry S. Battles. All authors have read and approved the final version of this manuscript and agree to be accountable for all aspects of the work in ensuring that questions related to the accuracy or integrity of any part of the work are appropriately investigated and resolved. All persons designated as authors qualify for authorship, and all those who qualify for authorship are listed.

## CONFLICT OF INTEREST

None declared.

## FUNDING INFORMATION

No funding was received or provided by the authors for the completion of this manuscript.

## Supporting information



Supporting Information

Supporting Information

## Data Availability

The raw data and processing pipeline for this article are available at the following address: https://github.com/Battles186/DecathlonCareerBest.git.
