# Supplementary material for: Predicting high‐performance decathlon career best
Source: Exp Physiol. 2025 Jan 9;110(11):1672–81. doi: 10.1113/EP091921 (PMC12576022; doi:10.1113/EP091921)
Supplement: Supplementary file 1 — Supporting Information [file EPH-110-1672-s002.docx]

# Supporting Information

The following is a summary of the raw, unmodified data provided in DecData.csv in the data deposition. This data was subsequently filtered to select eligible athletes, whose marks were then transformed, scaled, and analyzed as described in “Methods”.

“Competitor” through “Men 110h”:

|  | Competitor | Men.100 | Men.110h |
| --- | --- | --- | --- |
|  | Length:12762 | Min. :10.12 | Min. :13.36 |
|  | Class :character | 1st Qu.:11.05 | 1st Qu.:14.79 |
|  | Mode :character | Median :11.25 | Median :15.20 |
|  | NA | Mean :11.26 | Mean :15.26 |
|  | NA | 3rd Qu.:11.46 | 3rd Qu.:15.66 |
|  | NA | Max. :14.37 | Max. :23.55 |

“Men 400” through “Men HJ”:

|  | Men.400 | Men.LJ | Men.HJ |
| --- | --- | --- | --- |
|  | Min. :45.00 | Min. :4.140 | Min. :1.510 |
|  | 1st Qu.:49.67 | 1st Qu.:6.660 | 1st Qu.:1.850 |
|  | Median :50.66 | Median :6.900 | Median :1.920 |
|  | Mean :50.76 | Mean :6.899 | Mean :1.915 |
|  | 3rd Qu.:51.73 | 3rd Qu.:7.140 | 3rd Qu.:1.980 |
|  | Max. :59.67 | Max. :8.450 | Max. :2.280 |

“Men PV through Men’s DT”

|  | Men.PV | Men.SP | Men.DT |
| --- | --- | --- | --- |
|  | Min. :2.550 | Min. : 7.26 | Min. : 2.00 |
|  | 1st Qu.:4.110 | 1st Qu.:11.81 | 1st Qu.:35.02 |
|  | Median :4.400 | Median :12.80 | Median :38.43 |
|  | Mean :4.387 | Mean :12.82 | Mean :38.44 |
|  | 3rd Qu.:4.650 | 3rd Qu.:13.82 | 3rd Qu.:41.84 |
|  | Max. :5.600 | Max. :17.78 | Max. :55.87 |

“Men JT” through “Overall Score”:

|  | Men.JT | Men.1500 | Overall.Score |
| --- | --- | --- | --- |
|  | Min. :12.10 | Length:12762 | Min. :6400 |
|  | 1st Qu.:47.23 | Class :character | 1st Qu.:6766 |
|  | Median :51.86 | Mode :character | Median :7152 |
|  | Mean :52.10 | NA | Mean :7207 |
|  | 3rd Qu.:56.89 | NA | 3rd Qu.:7582 |
|  | Max. :79.05 | NA | Max. :9126 |

Raw marks for the men’s 1500m race are provided by World Athletics in character format (“4:36.7”, for example). Therefore, they are represented here as text rather than as numerical values.
